# Precision dual-comb spectroscopy using wavelength-converted frequency combs with low repetition rates

**DOI:** 10.1038/s41598-023-29734-2

**Published:** 2023-02-13

**Authors:** Yohei Sugiyama, Tsubasa Kashimura, Keiju Kashimoto, Daisuke Akamatsu, Feng-Lei Hong

**Affiliations:** Department of Physics, Graduate School of Engineering Science, Yokohama National University, 79-5 Tokiwadai, Hodogaya-Ku, Yokohama, 240-8501 Japan

**Keywords:** Optical spectroscopy, Mode-locked lasers

## Abstract

Precision spectroscopy contributed significantly to the development of quantum mechanics in its early stages. In the twenty-first century, precision spectroscopy has played an important role in several fields, including fundamental physics, precision measurement, environmental monitoring, and medical diagnostics. An optical frequency comb is indispensable in determining the frequency axis in precision spectroscopy and it is useful as a light source for spectroscopy. Dual-comb spectroscopy uses two frequency combs with slightly different repetition rates and has the potential to surpass conventional Fourier-transform infrared spectrometers. The resolution of dual-comb spectroscopy is limited by the frequency spacing of the comb components, that is, the repetition rate of the comb. We demonstrate dual-comb spectroscopy in the visible-wavelength region using wavelength-converted frequency combs from Er-doped fiber combs. The repetition rates of the combs are relatively low at 19.8 MHz, resulting in relatively high resolution in the dual-comb spectroscopy. The observed spectral shape in dual-comb spectroscopy agrees well with the fitting result based on the hyperfine structure of molecular iodine. The realized dual-comb spectroscopy using wavelength-converted Er-doped fiber combs is reliable (maintenance free) and applicable in other experiments at visible wavelengths.

## Introduction

An optical frequency comb^1,2^, generally produced by a mode-locked laser, is represented as a group of frequency components with equal spacing in the frequency axis and ultrashort optical pulses in the time axis. Optical frequency combs are extensively used in frequency metrology^3^, environmental monitoring^4^, medical diagnostics^5^, material characterization^6^, and other research fields. Although optical frequency combs are mainly used as a frequency measurement tool based on one comb component, they have recently been used as a light source involving a group of comb components. For instance, dual-comb spectroscopy (DCS) uses two broadband combs with slightly different repetition rates to observe the absorption and dispersion characteristics of various materials such as molecular gas^7,8^. DCS has the potential to surpass conventional Fourier-transform infrared spectrometers in several laboratory and field applications^9^.

In DCS, spectroscopic resolution is limited by the frequency spacing of the comb components, which is the repetition rate of the combs. The limited resolution can be improved by scanning the comb components^10,11^. However, changes in the environmental conditions may occur during scanning. Therefore, it is preferable to use frequency combs with a low repetition rate in high-precision DCS. Optical frequency combs based on mode-locked Er:fiber lasers (Er:fiber combs) are compact, robust, cost efficient, and typically operate at a repetition rate of 50–100 MHz. Herein, we developed Er:fiber combs using a nonlinear-polarization-evolution mode-locking scheme^12,13^ with a repetition rate of approximately 19.8 MHz^14^. Precision DCS of acetylene was performed using the developed low-repetition-rate combs. The normalized spectra of the P- and R-branches of the ν_1_ + ν_3_ vibration band of ^12^C_2_H_2_ were observed at a comb repetition rate of 19.8 MHz^15^. Instantaneous pressure changes in the cell were also observed during cooling of the liquid nitrogen trap^15^.

DCS is well established in the infrared and near-infrared regions because: 1) most comb light sources are in the near-infrared region; and 2) there is a ‘fingerprint region’ of molecules in the infrared region. In addition to the molecular fingerprint in the infrared region, molecules also exhibit several strong transitions in the visible region. However, there have been very few research activities related to DCS in the visible region. The first visible DCS was performed using frequency-doubled Yb:fiber combs at 520 nm to observe the absorption lines of molecular iodine at a spectroscopic resolution of 100 MHz^16^. DCS was performed using frequency-doubled Er:fiber combs at approximately 775 nm to observe the spectra of Rb and O_2_^17^. DCS has not yet been implemented using frequency-tripled combs. Because the Er:fiber comb has advantages over the Yb:fiber comb in terms of reliability (long-term operation and maintenance-free characteristics) and cost efficiency^13,18^, it is important to demonstrate visible DCS using frequency-tripled Er:fiber combs.

In this study, we generated visible combs at 532 nm by tripling the frequency of Er:fiber combs using MgO-doped periodically poled lithium niobate (PPLN) crystals. DCS is performed for molecular iodine with a spectroscopic resolution of 19.8 MHz using the visible combs. Data analysis was carefully performed to avoid aliasing in the spectral analysis^8,19,20^, which is particularly important when low-repetition-rate combs are used. Spectral normalization of DCS was performed based on the reference spectrum obtained by trapping molecular iodine in the cold finger using liquid nitrogen. The observed dual-comb spectrum can be reproduced using theoretical fitting based on Doppler-free results of the hyperfine structure of molecular iodine. The realized dual-comb spectroscopy using wavelength-converted Er-doped fiber combs is reliable (maintenance free) and applicable in other experiments at visible wavelengths.

## Results

### Frequency tripling of the Er:fiber combs

**Figure 1:**
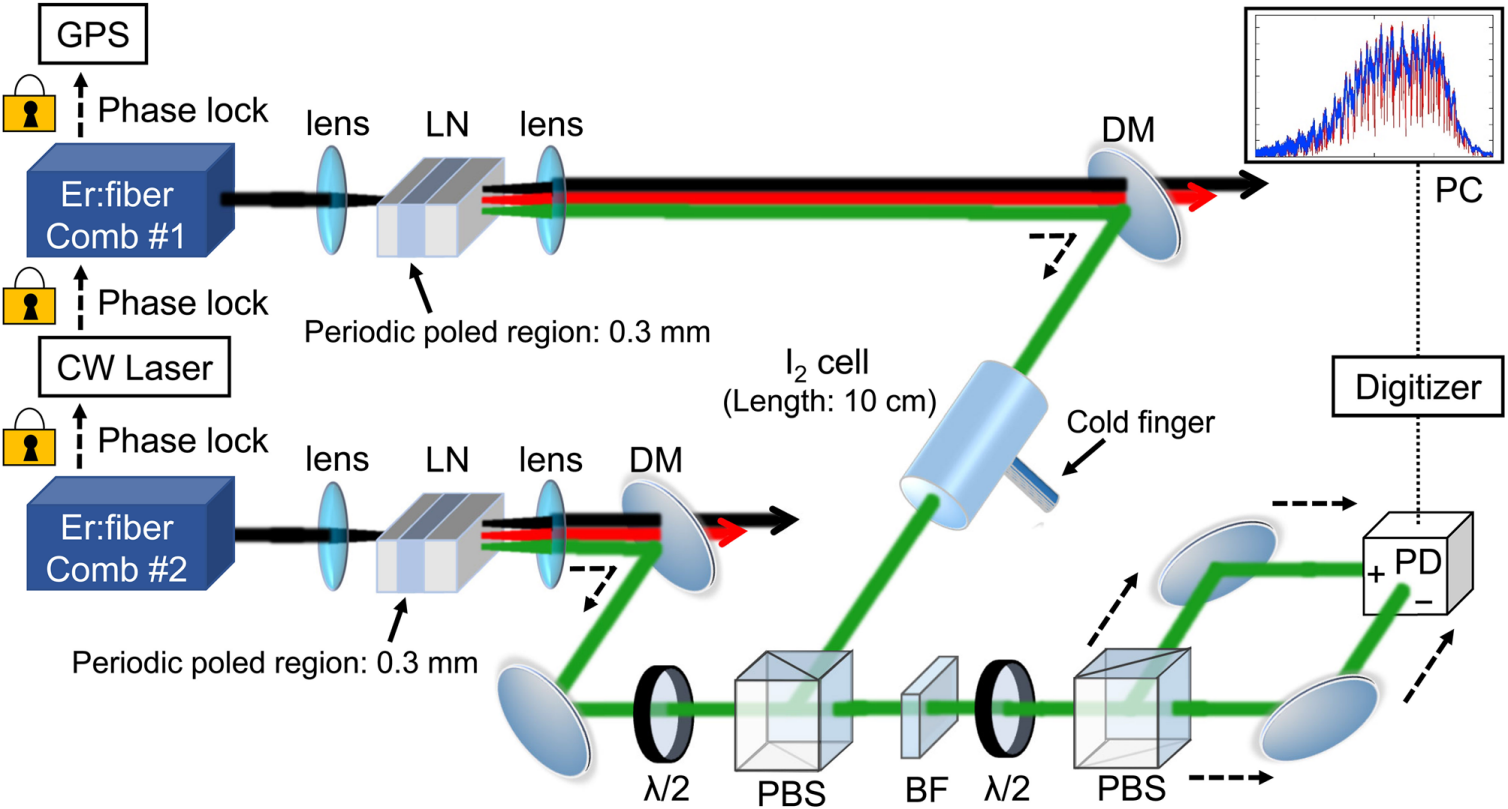
Schematic of the experimental setup. *GPS* global positioning system, *CW* continuous wave, *LN* lithium niobate, *DM* dichroic mirror,$\lambda /2$ half-wave plate, *PBS* polarizing beam splitter, *BF* bandpass filter, and photodetector.

Figure 1 illustrates a schematic of the experimental setup for the Er:fiber combs, wavelength conversion, and DCS. We describe the details of the setup in the Methods section. Briefly; the two Er:fiber combs (#1 and #2) had repetition frequencies (${f}_{\mathrm{rep}}$) of approximately 19.8 MHz^14^ and were self-referenced^2^. Comb #2 was phase-locked to comb #1 using a continuous wave (CW) laser with a broad servo bandwidth. Consequently, a small relative linewidth (≤ 1 Hz) between combs #1 and #2 was realized using the obtained broad servo bandwidth. During the phase-lock, the difference in the repetition rates (${\Delta f}_{\mathrm{rep}}$) between combs #1 and #2 can be set such that it is greater than the relative linewidth. Er:fiber combs have multiple output branches for different applications. The output branch used in combs #1 and #2 was equipped with an erbium-doped fiber amplifier (EDFA). The output beam of both combs was collimated with a diameter of approximately 860 µm and focused to 12 µm using an aspheric lens. The focused beam was injected into a lithium niobate (LN) crystal for frequency tripling. The crystal contained a PPLN region (pole length of 0.3 mm) with a chirped structure (HC Photonics). Because the PPLN was designed to triple the laser frequency via second-harmonic generation (1560 nm → 780 nm) and sum-frequency generation (1560 nm + 780 nm → 520 nm), the wavelength-converted laser beams contain fundamental, frequency-doubled, and frequency-tripled combs. The wavelength-converted laser beams were collimated using another aspheric lens and reflected by a dichroic mirror (DM) to separate the frequency-tripled comb from the fundamental and frequency-doubled combs. The frequency-tripled comb from comb #1 (used as the signal comb in the DCS of molecular iodine) was transmitted through an iodine cell with a length of 10 cm. In contrast, the frequency-tripled comb from comb #2 was used as the local comb in the DCS. The signal and local combs were overlapped using a polarizing beam splitter. After passing through a bandpass filter (BF), which was used to avoid aliasing (discussed in the next section), the beams were again divided into two beams and detected using two GaAs detectors for balanced detection^19^. The detected signal, which is an interferogram of the signal and local combs, was guided to a 14-bit digitizer for data analysis.

**Figure 2:**
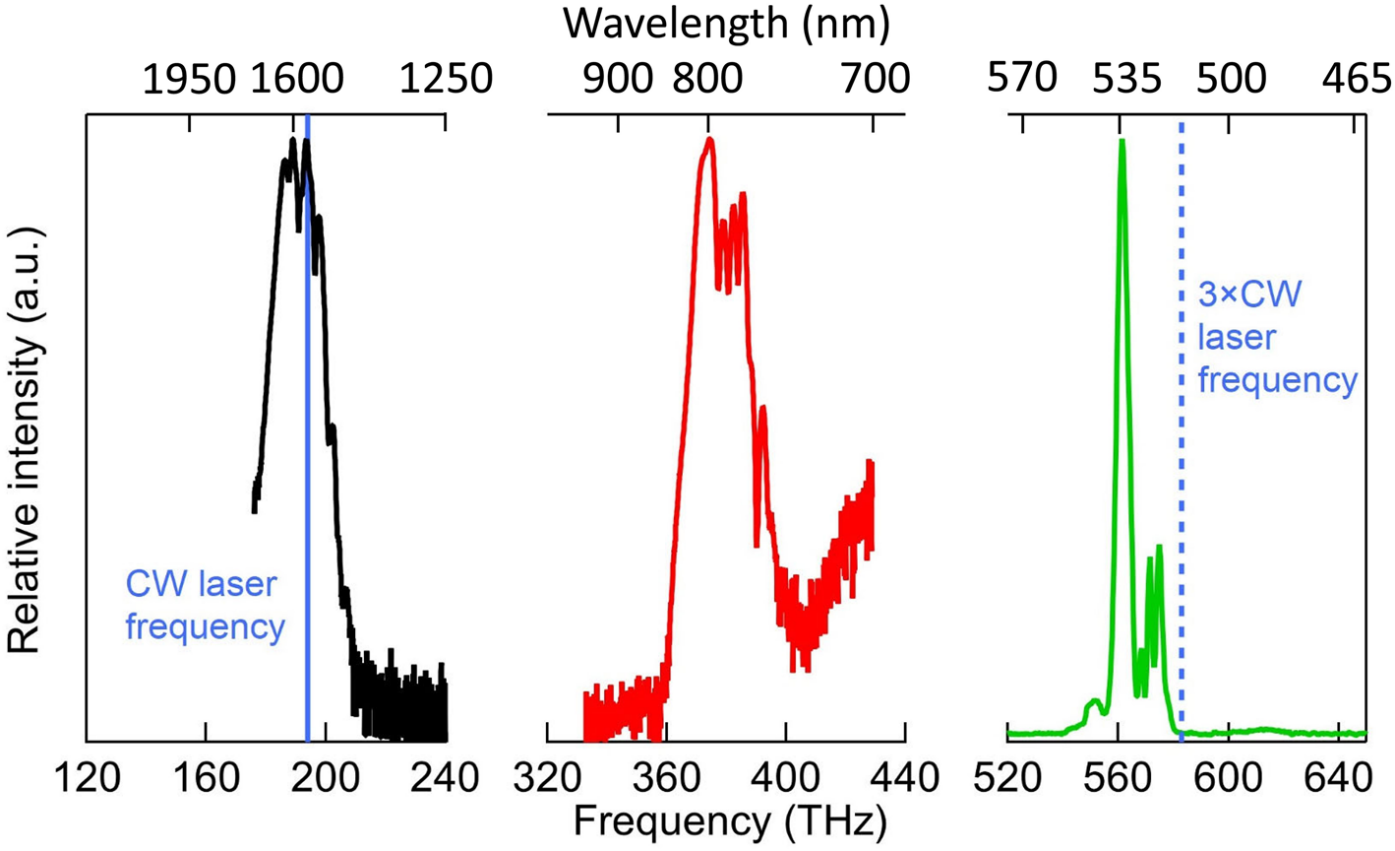
Spectrum of the original (black solid curve at approximately 1570 nm), frequency-doubled (red solid curve at approximately 792 nm), and frequency-tripled (green solid curve at approximately 534 nm) combs. The vertical scales of the spectrum in different wavelength regions are normalized to have the same maximum value. The blue solid line represents the frequency (194.4 THz, 1542 nm) of the CW laser for locking the signal and local combs. The blue dashed line represents the frequency of 3 times the CW laser frequency.

Figure 2 shows the spectra of the fundamental (black curve), frequency-doubled (red curve), and frequency-tripled (green curve) combs of comb #1. The fundamental and frequency-doubled combs were measured on the beams transmitted after the DM, using an optical spectrum analyzer (Agilent 86145 B). The frequency-tripled comb was measured on the beam reflected by the DM using a visible spectrometer (Ocean Optics USB2000+). The vertical scales of the spectra in different wavelength regions were normalized to obtain the same maximum value. The fundamental comb generated by the EDFA in the branch of comb #1 has a spectral bandwidth of approximately 115 nm, at approximately 1570 nm. The frequency-doubled (frequency-tripled) comb has a spectral bandwidth of approximately 54 (4) nm, at approximately 792 (534) nm. The observed center of the frequency-tripled comb (534 nm) was different from the designed center wavelength of 520 nm and was obtained by changing the phase-matching condition (the incidence angle of the laser beam into the crystal). The side lobes in the frequency-tripled comb spectrum were removed by the BF used in the DCS spectrometer. In this experiment, the center wavelength of 534 nm was selected because it is close to 532 nm, where Doppler-free spectroscopy of molecular iodine^21,22^ was performed, and the results can be used for comparison. We confirmed that the frequency-tripled combs of comb #2 also contain comb components in the 532 nm wavelength region.

The blue solid line in Fig. 2 shows the wavelength (frequency) of the CW laser used for phase locking the combs. We note that the tripled frequency of the CW laser (blue dashed line) is slightly outside the frequency region of the frequency-tripled comb.

### Data analysis method to avoid aliasing

The data analysis method to avoid aliasing in DCS has been discussed in literatures^8,19,20^. For DCS using low-repetition-rate combs and in the case where the CW laser for locking is outside the comb spectral rage, some detailed explanations for data analysis are needed.

The ${f}_{\mathrm{rep}}$ values of the signal comb (${f}_{\mathrm{rep},\mathrm{ S}}$) and local comb (${f}_{\mathrm{rep},\mathrm{ L}}$) in DCS are slightly different. Assuming that ${f}_{\mathrm{rep},\mathrm{ S}}$ is greater than ${f}_{\mathrm{rep},\mathrm{ L}}$ by ${\Delta f}_{\mathrm{rep}}$, we obtain:

1 $${f}_{\mathrm{rep},\mathrm{ S}} ={ f}_{\mathrm{rep},\mathrm{ L}} + {\Delta f}_{\mathrm{rep}}.$$

**Figure 3:**
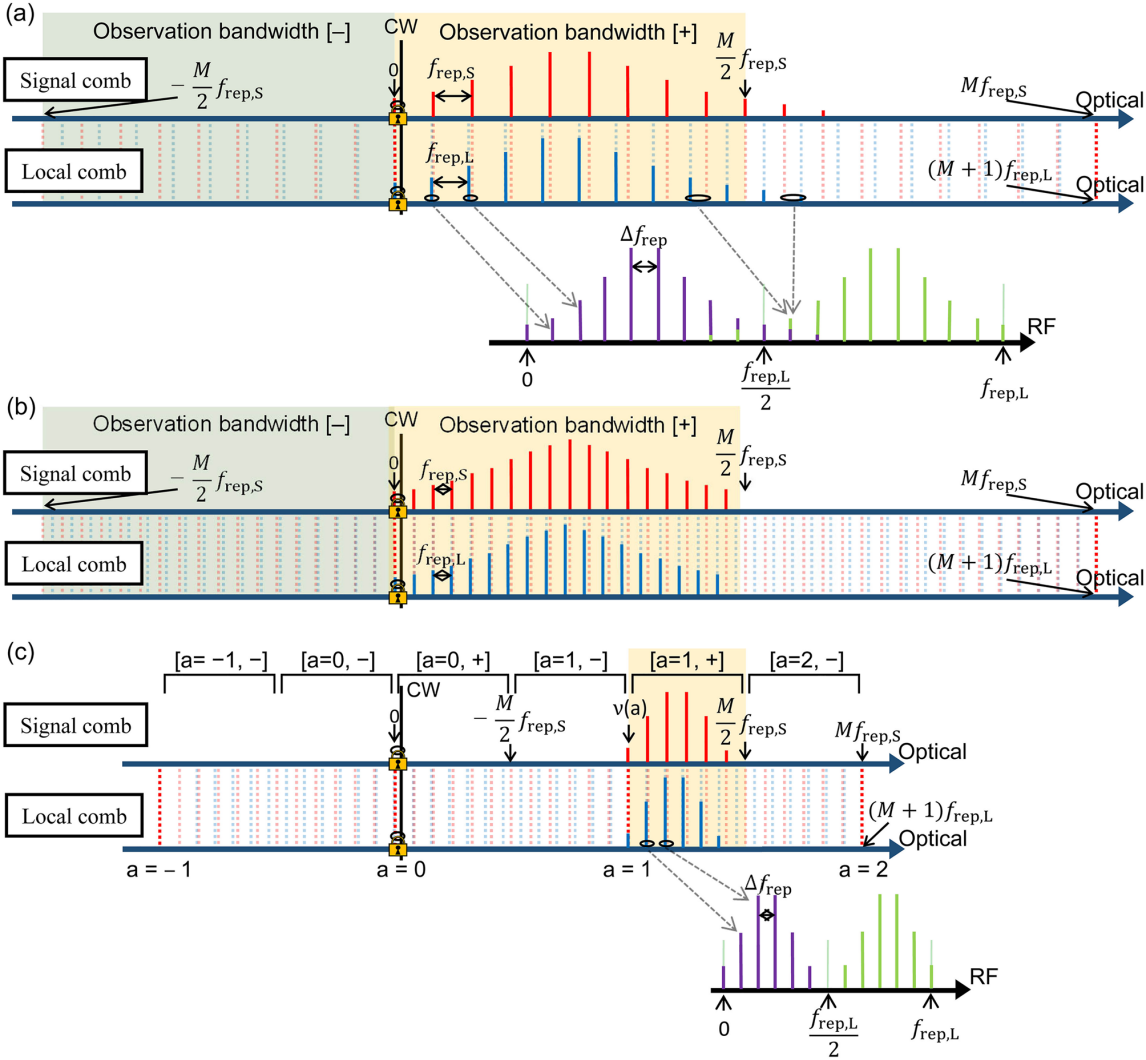
Observation bandwidth and the frequency relationship of the CW laser, signal, and local combs in DCS. (**a**) The observation bandwidth is around the CW laser and the first coincident comb components. The resulting RF comb is also shown with one-to-one correspondence from the signal and local combs. (**b**) The observation bandwidth obtained with low-repetition-rate combs. To achieve the same observation bandwidth as in (**a**), ${\Delta f}_{\mathrm{rep}}$ is decreased and *M* is increased compared to the (**a**) case. (**c**) A method to use the observation bandwidth that is set away from the CW laser and the first frequency coincident components. Using this method, one can set an appropriate observation bandwidth with optimal spectral resolution, ${\Delta f}_{\mathrm{rep}}$, measurement time, and data points.

Figure 3a shows the frequency relationship of the CW laser, signal, and local combs, along with the resulting radio frequency (RF) beat signals in the DCS. In the phase-locking process, the frequency relationship between the combs is fixed based on the frequency arrangement of the locking between the CW laser and the combs. For instance, one can lock the beat frequency between the CW laser and the nearest component of the signal comb at a fixed frequency (${f}_{\mathrm{beat}}$) and lock the local comb using the exact same method and frequency. Therefore, the comb components nearest to the CW laser in the signal and local combs have the same optical frequency. The two components belonging to the signal and local combs are frequency-coincident. We note that depending on the frequency arrangement of the locking between the CW laser and the combs (for instance, using different signs for ${f}_{\mathrm{beat}}$), the coincident comb components are not necessary for the nearest comb components to the CW laser. Owing to the difference between ${f}_{\mathrm{rep},\mathrm{ S}}$ and ${f}_{\mathrm{rep},\mathrm{ L}}$, the *M*th component from the first frequency-coincident component in the signal comb is frequency-coincident with the (*M* + 1)th component in the local comb. This condition of frequency coincidence is expressed as follows:

2 $$M{f}_{\mathrm{rep},\mathrm{ S}}=\left(M+1\right){ f}_{\mathrm{rep},\mathrm{ L}} ,$$

where *M* denotes the ‘magic number’ in DCS. Using Eqs. (1) and (2), we obtain:

3 $$M = \frac{ {f}_{\mathrm{rep},\mathrm{ L}}}{{\Delta f}_{\mathrm{rep}}} .$$

When the signal and local combs interfere in DCS, the resulting beat notes of the two combs are equally spaced by ${\Delta f}_{\mathrm{rep}}$ in the RF domain (referred to as the RF comb). The molecular absorption lines observed on the signal comb were mapped one-to-one on the RF comb. When the RF comb frequency exceeded ${f}_{\mathrm{rep},\mathrm{ L}}/2$, more than two signal comb components were mapped on the same RF component (aliasing). To maintain one-to-one mapping between the signal and RF comb components, the RF comb frequency was limited to a maximum value of ${f}_{\mathrm{rep},\mathrm{ L}}/2$. Any signal beyond ${f}_{\mathrm{rep},\mathrm{ L}}/2$ was cut using a low-pass filter. Therefore, the corresponding observation bandwidth ($\Delta \nu$) in the optical domain can be expressed as follows:

4 $$\Delta \nu = \frac{M}{2}{f}_{\mathrm{rep},\mathrm{ S}}=\frac{{f}_{\mathrm{rep},\mathrm{ S}} {f}_{\mathrm{rep},\mathrm{ L}}}{2{\Delta f}_{\mathrm{rep}}}$$

This is referred to as the Nyquist condition^7,8^ and it is indicated by a yellow shaded area (at the high-frequency side of the coincident comb components, observation bandwidth [ +]) in Fig. 3a. We note that instead of the observation bandwidth [ +], one can also use the observation bandwidth [ −] (at the low-frequency side of the coincident comb components) for DCS, as shown by the green-shaded area in Fig. 3a. When we mapped the observed molecular absorption lines on the RF comb back to the optical domain in the data analysis, the optical frequency axis was determined based on the absolute frequency of the coincident components in the signal and local combs.

In DCS using low-repetition-rate combs, the observation bandwidth $\Delta \nu$ is usually less than that in the high-repetition-rate case, as expressed in Eq. (4). To increase $\Delta \nu$, ${\Delta f}_{\mathrm{rep}}$ must be quadratically decreased (*M* increases linearly). Figure 3b shows an image of the signal and local combs for achieving the same observation bandwidth as in Fig. 3a when the repetition rates of the combs are low. In this case, DCS can be performed at a higher resolution (owing to a lower repetition rate) and the same observation bandwidth. However, this improvement was obtained by considering the cost of the decrease in ${\Delta f}_{\mathrm{rep}}$. The disadvantages of reducing ${\Delta f}_{\mathrm{rep}}$ are as follows:

1. The ${\Delta f}_{\mathrm{rep}}$ must be significantly greater than the relative linewidth of the two combs. Otherwise, adjacent RF comb components cannot be fully resolved. For instance, DCS was performed across 1.0–1.9 μm (140 THz) using combs with a repetition rate of approximately 48 MHz and ${\Delta f}_{\mathrm{rep}}=7.6 \mathrm{Hz}$^23^. In this case, if the repetition rate is similar to the rate used in this experiment (19.8 MHz), ${\Delta f}_{\mathrm{rep}}$ must be set at 1.3 Hz, which is very close to the Hz-level relative linewidth between the combs.
2. In DCS, the observation time is inversely proportional to ${\Delta f}_{\mathrm{rep}}$. Usually, the averaging of measurements is necessary to increase the S/N of the observed spectra. Furthermore, it is necessary to perform DCS twice, not only for the sample placed in the signal comb path but also for the background spectra without a sample, to obtain spectral normalization^8,9^. The increase in the observation time may degrade the performance of the averaging and normalization owing to the variation in the background spectra during the measurements.
3. In DCS, the number of data points in the analysis is inversely proportional to ${\Delta f}_{\mathrm{rep}}$. A decrease in ${\Delta f}_{\mathrm{rep}}$ increases the number of data points, which is usually a large number in DCS, and increases the handling difficulty in data processing.

However, from the needs point of view, a small ${\Delta f}_{\mathrm{rep}}$ (large observation bandwidth) is not always required. In this experiment, the frequency-tripled comb had a relatively small spectral range limited by the phase-matching condition in the wavelength conversion (as shown in Fig. 2). Therefore, DCS is only required to cover the spectral range of the frequency-tripled comb. Furthermore, if the atomic or molecular spectrum of interest is within a narrow spectral region, a large observation bandwidth is not required. Therefore, it is important to set the required observation bandwidth in the frequency region of interest with an appropriate ${\Delta f}_{\mathrm{rep}}$, according to the comb repetition rate and other experimental conditions, such as the relative linewidth, measurement time, and data points.

Figure 3c indicates an example to meet these requirements. A relatively small observation bandwidth is set at a spectral region, which is away from the first frequency coincident components (referred to as ‘$a$ = 0’). To meet the Nyquist condition, the spectral region of this observation bandwidth satisfies Eq. (4); however, it is in a region beyond the conventional observation bandwidths of [$a$= 0, +] and [$a$= 0, −]. In Fig. 3c, the observation bandwidth is set at the high-frequency side ( +) of the second frequency coincident components ($a$= 1) and referred to as [$a$= 1, +]. The observation bandwidth is typically set using an optical BF in a dual-comb spectrometer. The RF comb resulting from DCS is low-pass filtered at ${f}_{\mathrm{rep},\mathrm{ L}}/2$ to fulfill the Nyquist condition. For a one-to-one mapping of the observed absorption lines on the RF comb back to the optical domain, the optical frequency axis must be determined using the absolute frequency of the coincident components as follows:

5 $$\nu \left(a\right) = \left({\nu }_{\mathrm{CW}}- {f}_{\mathrm{beat}}\right) + aM{f}_{\mathrm{rep},\mathrm{ S}},$$

where ${\nu }_{\mathrm{CW}}$ denotes the frequency of the CW laser used in the phase-lock, and the sign before ${f}_{\mathrm{beat}}$ is determined by the frequency relationship between the CW laser and the nearest components. We note that a similar equation as Eq. (5) has appeared in^19^, although no further development of the equation was found there. The assignment of the observation bandwidth [$a$, ±] is also used to calculate the optical frequency axis. Both the number ‘$a$’ and the sign ‘ ± ’ are determined by the rough estimation of the center of the observation bandwidth (usually the center frequency of the optical bandpass covering the observation bandwidth). In this way, the degrees of freedom for setting ${\Delta f}_{\mathrm{rep}}$ are drastically increased. Therefore, most problems associated with the setting of ${\Delta f}_{\mathrm{rep}}$, such as the relative linewidth, measurement time, and data points, can be avoided. With this method, we can set the observation bandwidth [$a$, ±] using any number ‘$a$’ and sign ‘ ± ’ across the optical frequency domain. Meanwhile, because the repetition rate of combs is relatively low, high-resolution DCS can be achieved.

### Iodine spectra observed using DCS

**Figure 4:**
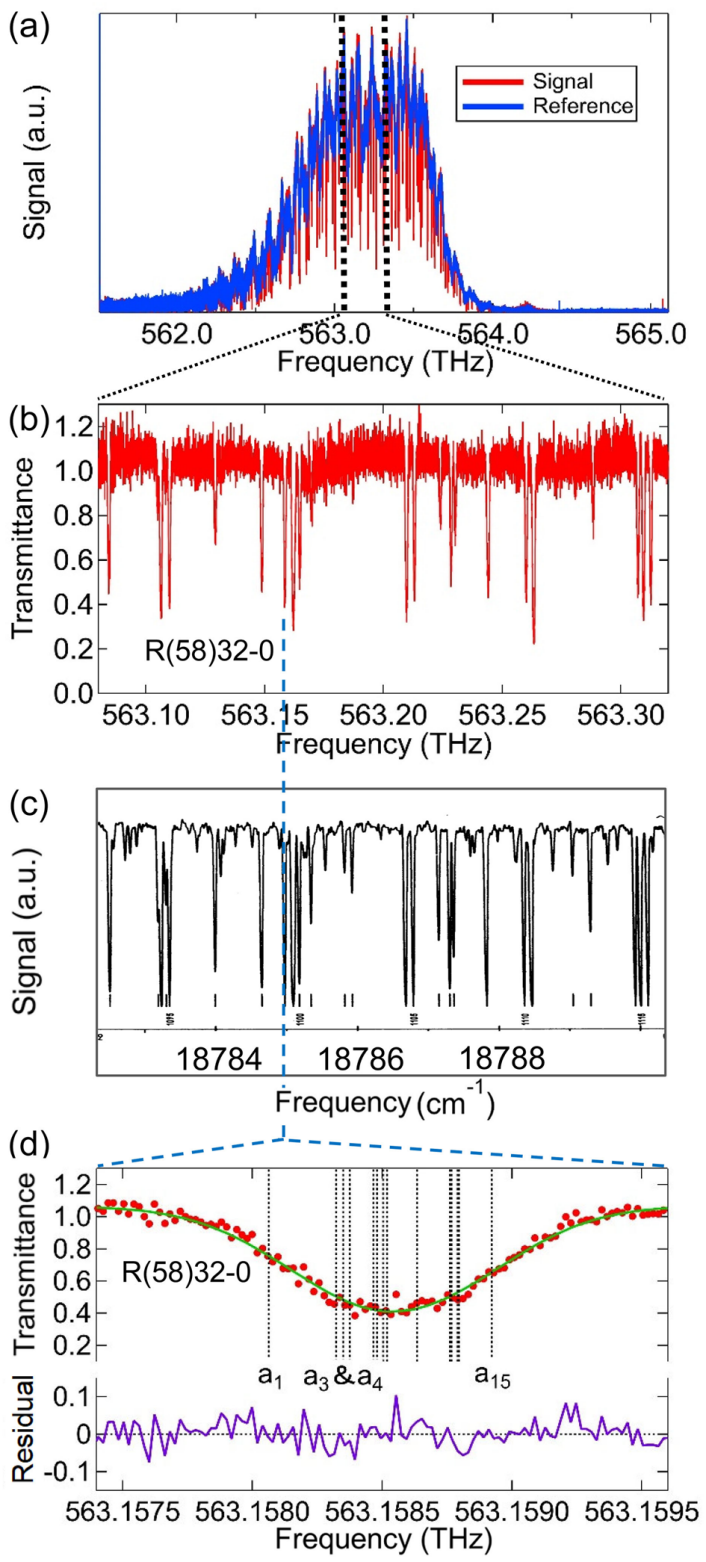
(**a**) Observed signal and reference spectra using DCS. The signal spectrum contains the absorption lines of ^127^I_2_, whereas the reference spectrum contains only background spectrum of the dual comb spectrometer without the gas sample of ^127^I_2_. (**b**) Normalized spectra obtained (**a**) in an enlarged frequency range of 563.08–563.32 THz. (**c**) Iodine spectra obtained using Fourier transform infrared spectroscopy^26^, which is shown here for comparison. The full range of the horizontal axis (18,782.3–18,790.3 ${\mathrm{cm}}^{-1}$) is equivalent to (**b**). (**d**) Enlarged view of the R(58)32-0 line (red solid circles) and the theoretical fit (green curve) by overlapping the Gauss profiles of 15 hyperfine components (shown as vertical lines). Fitting residual (purple curve) is also shown.

Figure 4a shows the observed signal (red curve) and reference (blue curve) spectra of the absorption lines of ^127^I_2_ in a frequency range of 561.6–565.2 THz. ${\Delta f}_{\mathrm{rep}}$ was set at 54 Hz. The calculated observation bandwidth $\Delta \nu$ based on Eq. (4) is 3.6 THz, which corresponds to the frequency range of the horizontal axis in Fig. 4a. The global shape of the spectrum was determined using an optical BF in the spectrometer. The center wavelength and bandwidth (full width at half maximum) of the BF were 532 nm (563.5 THz) and 1 nm (1 THz), respectively. The CW laser (RIO 0194-3-01-4-AT8) used to phase-lock the combs had a relatively narrow tuning bandwidth (0.03 nm) centered at 1542.38 nm and was used in the Doppler-free spectroscopy of the P(16) line of the *ν*_1_ + *ν*_3_ vibration band of ^13^C_2_H_2_^24^ in other experiments. The tripled frequency of the CW laser was 583.11 THz (514.13 nm), which significantly varies from the center frequency of the band pass filter (563.5 THz). The observation bandwidth shown in Fig. 4a is determined as [$a$ = − 3, +], which is the sixth window of the observation bandwidth at the lower frequency side from the first frequency coincident component. In this experiment, the beat frequencies between the CW laser and the nearest component of the signal and local combs (${f}_{\mathrm{beat}}$) were set at the same frequency (4 MHz) with the same sign. Therefore, the first frequency coincident components are the nearest comb components to the CW laser used in the phase locking.

In Fig. 4a, the blue curve is overwritten by the red curve. The signal spectrum (red curve) shows both iodine absorption lines and the background spectrum, which cannot be observed in the figure because they overlap with the background spectrum in the reference spectrum (blue curve). When the signal spectrum was measured, the pressure of the molecular iodine in the cell was approximately 26 Pa. This is the saturated vapor pressure of molecular iodine at 23 ℃, the temperature of the cell cold finger (same as room temperature in this case). Before the reference spectrum was measured, liquid nitrogen was used to trap molecular iodine in the cold finger. By cooling the cold finger of the cell to a liquid nitrogen temperature of − 196 ℃ (77 K), the gas pressure in the cell becomes infinitely close to zero^25^. The only difference between the signal and reference spectra is the presence or absence of an absorber in the cell (molecular iodine gas). Therefore, the normalized spectrum (transmittance) of molecular iodine can be obtained by dividing the signal spectrum by the reference spectrum. We note that liquid nitrogen trap was previously used in acetylene DCS experiment for spectral normalization^15,26^. The fringe in the global shape of the spectrum originates mainly from the etalon effects of the BF. This fringe effect can also be eliminated in spectral normalization. The minimum time needed to record the signal or reference spectrum was 18.5 ms, determined using a ${\Delta f}_{\mathrm{rep}}$ of 54 Hz. The total number of data points of the signal or reference spectrum was 0.18 million. The spectra illustrated in Fig. 4a were obtained by averaging the interferograms over 30,000 times for 556 s.

Spectral normalization was performed using the observed signal and the reference spectra, as shown in Fig. 4a. Figure 4b shows the enlarged view of the normalized spectrum in the range of 563.08–563.32 THz. Approximately 20 relatively strong iodine lines are observed in this frequency range. Most of the lines were studied using Doppler-free spectroscopy and are recommended by the International Committee for Weights and Measures (CIPM) as optical frequency references^27^. The S/N of the R(58)32–0 line was approximately 8.4, which is to be compared with the S/N of approximately 30 reported in a previous study^16^. The S/N ratio in this experiment was mainly limited by the low optical power and short iodine cell length. The low optical power at 532 nm (0.54 mW in this experiment) was due to the phase-matching condition of the tripling crystal. The obtained center of the frequency-tripled comb in this experiment was 534 nm, and the power at 532 nm was significantly less. The optical power can be improved by fabricating a crystal with the proposed that targets 532 nm light. We note the tripled power of an Er:fiber comb at 532 nm is usually smaller that the doubled power of a Yb:fiber comb. Therefore, the disadvantage of the frequency-tripled Er:fiber comb over the frequency-doubled Yb:fiber comb is the obtained small optical power that limits the S/N of the spectrum. The advantage is the reliability (long-term operation and maintenance-free characteristics) of the Er:fiber comb. The iodine cell used in a previous study^16^ was 90 cm, 9 times longer than that of the cell in this experiment. To achieve a better S/N, we averaged the interferograms over 30,000 times for 556 s, whereas in a previous study^16^, the spectrum was measured within 12 ms (without averaging). With a long measurement time, the background spectrum may change owing to the temperature effects on the BF and/or the laser oscillator. The center of the baseline of the normalized spectrum (transmittance) in Fig. 4b is not exactly 1, indicating a background spectral change between the signal and reference spectra. Furthermore, the fringe-like variation in the baseline was caused by the background spectrum change. Therefore, a reduction in the background spectrum change is necessary to further increase the S/N ratio by averaging. This can be done by controlling the temperature of the laser oscillator and key optical components, such as the BF. Of course, if the short-tem S/N can be improved by increasing the optical power of the tripled comb, the average over long time and hence temperature control become unnecessary. For comparison, Fig. 4c shows the iodine spectra obtained using Fourier transform infrared spectroscopy, as published in the iodine atlas^28^. Figure 4b,c show the identical iodine spectra in the same frequency range.

Figure 4d shows an enlarged view of the R(58)32–0 line (solid red circles) across 2 GHz. The resolution of DCS is 19.8 MHz and there are approximately 100 data points in this frequency range. For comparison, we note that in a previous study^16^, the resolution of DCS was 100 MHz, and there were approximately 20 data points in the same frequency range. The R(58)32–0 line has been previously investigated using saturation spectroscopy^29^. This line contains 15 hyperfine components across 858 MHz^29^. The optical frequency of each hyperfine component can be determined using the measured hyperfine splittings^29^ and the absolute frequency of the a_1_ component of the line^30^, with an uncertainty at the kHz level. We also indicate the frequency positions of the hyperfine components using the vertical lines in Fig. 4d. In this experiment, each hyperfine component was Doppler-broadened at the GHz level. Pressure broadening is at the MHz level and can be ignored. Finally, we fitted the observed DCS signal using a line profile (green curve), which was an overlap of 15 Gaussian profiles with hyperfine splittings as frequency intervals. The residual of the fit is also shown in Fig. 4d by the purple curve, which is approximately 3.5 × 10^−2^ in standard deviation. The fitted line center was − 13(4) MHz from the weighted line center calculated using the measured hyperfine splittings^29^ and the absolute frequency of the a_1_ component of the line^30^.

## Discussion

In DCS, the optical frequency axis is calibrated based on the absolute frequency of the coincident components, $\nu \left(a\right)$, which is determined using Eq. (5). According to Eq. (5), not only coefficients ‘$a$’ and ‘$M$’ are necessary but also the absolute frequency of the CW laser ${\nu }_{\mathrm{CW}}$ is required. In the phase-locking scheme for the signal and local combs, the CW laser is phase-locked to the signal comb, which is phase-locked to the global positioning system (GPS) frequency reference. Therefore, the remaining information for the calculation is the ‘*n*’ number of the signal comb component (*n*th comb component counted from the origin of the frequency axis) used in the phase locking. In the low-repetition-rate comb case, a careful procedure is required to determine the *n* because the absolute frequency of the CW laser needs to be measured independently with an uncertainty well below the comb repetition rate. There are four methods for determining *n*.

1. The frequency of the CW laser is independently determined using an optical frequency reference close to that of the CW laser. For instance, the P(16) line of the *ν*_1_ + *ν*_3_ vibration band of ^13^C_2_H_2_^25^ can be used as a frequency reference in this case. This can be achieved by measuring the beat frequency between the phase-locked CW laser and acetylene-stabilized laser.
2. The frequency of the phase-locked CW laser was measured using a wavemeter. In this case, the wavemeter needs to have a resolution higher than the comb repetition rate and be well calibrated with an uncertainty less than the comb repetition rate.
3. The frequency of the phase-locked CW laser was measured using another frequency comb with different repetition rates. The frequency of the CW laser was measured using two combs with significantly different repetition rates. Using this method, *n* can be unmistakably determined.
4. The frequency information of the measurement results was used. If we know the frequency of the measurement results in advance with an uncertainty less than the comb repetition rate, this information can be used to determine *n*.

In this experiment, we used methods 3 and 4 to determine *n* and calculate the frequency axis of the DCS results. In method 3, the CW laser was measured using two combs with repetition rates of 19.8 and 49 MHz. In method 4, Doppler-free data^29^ were used to determine *n*.

The nonlinear crystal used for tripling the frequency of the comb was a PPLN crystal targeting 520 nm and with a limited phase-matching wavelength range. For different applications using different wavelengths, the crystal must be redesigned and refabricated. In contrast, spectral broadening across a large wavelength range has been realized using PPLN waveguides. For instance, ultrabroadband combs covering up to four octaves in the visible to mid-infrared region have been generated in PPLN waveguides using Er:fiber combs^31–33^. Such broadband combs can be filtered out at the targeted wavelength and applied to DCS. The present method is particularly effective in such cases because the observation bandwidth can be set at an arbitrary wavelength with a proper bandwidth.

DCS using low-repetition-rate combs along with the data analysis method proposed in this study should expand the applications using DCS. For instance, the observed Doppler profile of acetylene absorption lines was used to derive the pressure broadening and pressure-shift coefficients based on a fit to a Voigt function^34^. DCS using low-repetition-rate combs has already exhibited smaller residuals and uncertainties of the fitting parameters^15^ compared to those reported in a previous study^34^. With an appropriate setting of the observation bandwidth, and consequently a smaller observation time, one may reduce the background spectrum change and further improve the DCS spectra. With such an improvement in the spectrum, better fitting results can be achieved. We also demonstrated instantaneous pressure changes inside the acetylene cell during cooling of the liquid nitrogen trap in a previous study^15^. The minimum measurement time was 26.7 ms, limited by a ${\Delta f}_{\mathrm{rep}}$ of 37.5 Hz. The observation bandwidth was 1524–1542 nm, obtained using a BF centered at 1530 nm, with a bandwidth of 12 nm^15^. The observation bandwidth can be further reduced to 1/10 by using a BF with a bandwidth of approximately 1.2 nm. Therefore, we should be able to increase ${\Delta f}_{\mathrm{rep}}$ by a factor of 10 and consequently reduce the measurement time by a factor of 10 to the millisecond level.

In conclusion, we developed two low-repetition-rate visible combs at approximately 532 nm for DCS by tripling the frequency of Er:fiber combs. DCS was performed for molecular iodine with a spectroscopic resolution of 19.8 MHz using the visible combs. The observed dual-comb spectrum was well reproduced using theoretical fitting based on the hyperfine structure of the molecular iodine. The realized dual-comb spectroscopy using wavelength-converted Er-doped fiber combs is reliable (maintenance free) and applicable in other experiments at visible wavelengths.

## Methods

### Experiment setup

Figure 1 shows a schematic of the experimental setup for the Er:fiber combs, wavelength conversion, and DCS. The laser oscillators of the signal and local combs are based on a nonlinear polarization evolution mode-lock scheme^12,13^. The configurations of the signal and local combs are similar, except that the local comb contains a delay line and an additional electro-optic modulator (EOM) in the laser cavity. The delay line was used to precisely adjust ${f}_{\mathrm{rep}}$ and set the repetition rate (${\Delta f}_{\mathrm{rep}}$) of the signal and local combs depending on the experimental situation. The ${f}_{\mathrm{rep}}$ of the signal comb (${f}_{\mathrm{rep},\mathrm{ S}}$) was stabilized to a GPS reference using a piezoelectric transducer in the laser cavity for a slow servo (servo bandwidth at the kHz level). A beat note (${f}_{\mathrm{beat},\mathrm{ S}}$) between the signal comb components and CW laser was detected and used to stabilize the frequency of the CW laser. The error signal of the ${f}_{\mathrm{beat},\mathrm{S}}$ servo, was fed back to the injection current of the CW laser for a relatively fast servo (servo bandwidth of 450 kHz). A beat note (${f}_{\mathrm{beat},\mathrm{ L}}$) between the local comb components and CW laser was detected and stabilized by controlling ${f}_{\mathrm{rep}}$ of the local comb (${f}_{\mathrm{rep},\mathrm{ L}}$) using the EOM for a relatively fast servo (servo bandwidth of 380 kHz).

## Data Availability

The data supporting the findings of this study are available from the corresponding author upon request.
